# Decoding the trends and the emerging research directions of E-commerce and tourism in the light of Resource Dependence Theory: A bibliometric analysis

**DOI:** 10.1016/j.heliyon.2024.e28076

**Published:** 2024-03-15

**Authors:** Mehmet Bahadır Kalıpçı, Erkan Kadir Şimşek, Ramazan Eren

**Affiliations:** a Department of Tourism and Travel Services, Akdeniz University, Antalya, Turkiye; b Department of Hotel, Restaurant and Catering Services, Akdeniz University, Antalya, Turkiye; c Tourism Management Department, Akdeniz University, Antalya, Turkiye

**Keywords:** e-commerce, Tourism, Resource dependence theory, Bibliometric analysis

## Abstract

E-commerce and tourism have all seen a lot of growth and development recently in both business and academia. The tourism sector has significantly changed because of the growth of e-commerce platforms, as more and more consumers buy their rooms and tickets online. The academics conducted further research to understand the impact of e-commerce platforms on the tourism industry, leading to the development of these two specific fields’ body of literature. A bibliometric review was carried out to draw the trends of the research conducted to date in the field of e-commerce and tourism. Thus, it is possible to have a general overview of the literature for academics to intent to conducting future research. Employing bibliometric analysis with Biblimetrix software on 456 publications from the Web of Science database covering the years from 2000 to 2022, this study identifies predominant themes and research trends in the field. The terms “e-commerce” and “tourism” were used as keywords. During the analysis, six research questions were answered and results were combined with Resource Dependence Theory. Australia and China which are the first two countries in terms of citations. China is the outstanding country for author collaborations. Co-citation network analysis identified four clusters, namely management, model, satisfaction, and quality. Publications with keyword “communication”, “enterprises”, “application” and “rural” are in a decrease after 2015. Finally, future research directions were proposed.

## 1 Introduction

In recent years, e-commerce and tourism have all experienced significant growth and development in both industry and academia [1]. The rise of e-commerce platforms has revolutionized the way that consumers shop and engage with businesses, creating new opportunities for entrepreneurs and transforming traditional industries such as retail and hospitality [2,3]. At the same time, the tourism industry has experienced rapid expansion, with more people than ever before traveling internationally and domestically for both leisure and business purposes. This growth has been fueled in part by the widespread adoption of online booking platforms and the increasing availability of information about destinations and experiences.

E-commerce and tourism are two distinct yet interconnected fields that have a significant impact on various aspects of modern society. With the rise of e-commerce platforms, the tourism industry has undergone a significant transformation, with more and more people using online platforms to book their travel accommodations and experiences [4]. This shift has also created new opportunities for bibliometric analysis, as researchers seek to understand the effect of online platforms for the tourism industry. Overall, the intersection of e-commerce, tourism, and bibliometrics presents a rich and complex area of study that has important implications for both academic research and practical applications in the tourism industry. As e-commerce continues to reshape the way we travel and engage with the world around us, the insights provided by bibliometric analysis will become increasingly valuable in understanding and responding to these changes. In academia, researchers are exploring the intersections of e-commerce, tourism, and bibliometrics, seeking to understand the complex relationships between these fields and to identify new areas of study and research [5]. This has resulted in the advancement of new theories, tools, and methods for analyzing online information and measuring the impact of e-commerce on the tourism industry.

Resource Dependence Theory is a widely used framework in organizational studies that explains how organizations are dependent on external resources to survive and thrive in their environment [6]. The theory posits that organizations must actively manage their relationships with external actors and institutions to secure the resources they need to achieve their goals [7]. In the context of e-commerce and tourism, Resource Dependence Theory suggests that businesses in the tourism industry are increasingly dependent on digital platforms and technologies to reach customers and compete in the marketplace [8]. As such, these businesses must carefully manage their relationships with online travel agencies, search engines, and other digital intermediaries to ensure they have access to the resources they need to succeed [9].

With the proliferation of e-commerce platforms, there has been a surge in the amount of online information available about tourist destinations, travel experiences, and accommodations [10]. Bibliometric analysis can help researchers identify the most important and influential sources of information, as well as track trends and changes in the field over time. Bibliometrics, the study of measuring and analyzing scholarly literature, has become increasingly important in the field of tourism [11]. However, bibliometric analysis can provide valuable insights into the trends and patterns of scholarly research related to these industries [12], such as studies on consumer behavior in e-commerce or destination marketing in tourism. Such insights can inform business strategies and marketing efforts, as well as academic research in these fields [13]. Additionally, bibliometric indicators such as citation counts and h-index scores may be used to evaluate the impact and visibility [14] of e-commerce and tourism research, which can influence funding and career advancement opportunities for scholars. In this way, bibliometrics indirectly affects the development and progress of e-commerce and tourism, by shaping the academic discourse and knowledge base that underpins these industries.

Drawing on Resource Dependence Theory, this study aims to explore the relationships between e-commerce, tourism, and bibliometrics, with a particular focus on how businesses and researchers can effectively manage their relationships with external actors and institutions to secure the resources they need to succeed. Because it can explain how organizations in the e-commerce and tourism industries rely on resources like technology, information, and networks, and how these dependencies influence their strategies and interactions, Resource Dependence Theory is the most appropriate framework for our research. Our research is driven by Resource Dependence Theory, which offers an organized method for recognizing and comprehending the connections between tourism and e-commerce with bibliometrics. By putting Resource Dependency Theory to use in the e-commerce and tourism industries. Therefore, the purpose of this study is to shed light on the methods and approaches organizations might employ to manage the intricate web of interactions that exists in the digital economy. Additionally, the study seeks to shed light on how bibliometric analysis can influence the research agendas and methods of scholars of tourism studies. Overarching goal of this study is to better understand the role of Resource Dependence Theory in shaping the relationships between e-commerce, tourism, and bibliometrics. By exploring how businesses and researchers can manage their dependencies on external resources, the study's goal is to contribute to a better understanding of the complicated dynamics that underpin these critical areas of inquiry. This research aims to comprehend how the outcomes of bibliometric analysis impact various management and marketing strategies in the tourism and e-commerce industries.

The significance of this research lies in its potential to offer crucial insights into the problems that tourism organizations may leverage to strengthen their e-commerce tactics, thereby improving consumer satisfaction and financial gains. These kinds of discoveries can be very helpful to scholars and practitioners alike.

## 2 Conceptual background

### 2.1 E-commerce (electronic commerce), tourism, and Resource Dependence Theory

The term “e-commerce” often known as “electronic commerce” describes the exchange of products and services over the internet [15] has revolutionized businesses' operations, allowing them to reach a wider audience and conduct transactions more efficiently. In recent years, e-commerce has seen explosive growth, with more and more consumers turning to online shopping as a convenient and accessible way to purchase goods. With the increasing prevalence of mobile devices and the rise of digital payment options, e-commerce is poised to continue its upward trajectory and transform the retail landscape [[16], [17], [18], [19]].

E-commerce has had a significant impact on the tourism industry, enabling travelers to plan and book their trips online [20]. It has made it easier for consumers to compare prices, read reviews, and make reservations, while also providing businesses with a cost-effective way to market their products and services [21]. E-commerce platforms have also enabled tourism businesses to reach a global audience, expanding their customer base beyond traditional geographic boundaries [22]. Additionally, e-commerce has facilitated the growth of new business models in the tourism industry, such as online travel agencies and vacation rental platforms, which have disrupted traditional brick-and-mortar travel agencies and hotels. Overall, e-commerce has transformed the way travelers plan and book their trips, while also creating new opportunities for tourism businesses to grow and innovate [23].

Tourism is a vital industry that contributes to economic growth and development worldwide. It involves the individuals moving from one location to another for the purpose of leisure, business, or other reasons [24]. Tourism can take many forms, from domestic travel to international trips, and encompasses a wide range of activities such as sightseeing, adventure sports, cultural events, and culinary experiences. The travel and tourism industry has seen major changes in last decades, with the increasing demand for new touristic destinations, the growth of sustainable tourism, and the impact of technology on travel. As such, it remains a dynamic and exciting field with numerous opportunities for businesses and travelers alike [25,26].

E-commerce and tourism are two industries that are closely related and have a significant impact on each other [27]. While tourism entails traveling for pleasure, business, or other reasons, e-commerce is the buying and selling of products and services through the internet [28]. The combination of these two industries has created a new trend in the way people plan and book their travel [20]. E-commerce has impacted the tourism sector in several ways, some of them are as follows. *Online Booking:* One of the most significant impacts of e-commerce on the tourism industry is the ability to book travel arrangements online. This has made it easier for people to plan their trips, compare prices, and book flights, hotels, and other services with just a few clicks [29,30]. *Increased Competition:* With the rise of e-commerce, there is an increased competition between travel and hospitality businesses in the tourism industry. This has led to better deals and packages for travelers, as businesses try to offer more value for money to attract customers [31]. *Personalization:* E-commerce has also allowed for greater personalization in the tourism industry. Travelers can now create custom itineraries and choose from a wide range of options to suit their preferences and budget [32,33]. *Customer Service:* With the use of e-commerce, businesses in the tourism industry can now offer better customer service, including online chat support and 24/7 assistance, which can help to build trust and loyalty with customers [34]. *Global Reach:* E-commerce has also given businesses in the tourism industry a global reach. They can now target customers from all over the world and offer their services to a wider audience, leading to increased revenue and growth opportunities [35,36]. Overall, the integration of e-commerce and tourism has brought about significant changes in the way people travel and plan their trips. It has made the process more convenient, personalized, and accessible, leading to increased growth and competition in the industry.

The convergence of e-commerce and tourism has been an increasingly popular area of research [37]. Scholars have recognized the potential for e-commerce to transform the way tourism businesses operate, while also enhancing the travel experience for consumers. As a consequence, there is a growing corpus of literature that explores the connection between e-commerce and tourism-related topics [30,[38], [39], [40], [41], [42]] in an effort to pinpoint the advantages and disadvantages of this convergence. The expansion of the scientific literature related to tourism and e-commerce also reveals the need to examine this field of study with an innovative approach.

Resource Dependence Theory (RDT) has been widely used in organizational studies to explain how organizations depend on external resources to achieve their goals [6]. The theory suggests that organizations must manage their relationships with external actors and institutions to ensure they have access to the resources they need to survive and thrive [43]. were the first to articulate RDT, arguing that organizations must engage in a variety of strategies to manage their dependencies on external resources. These strategies include building alliances, negotiating contracts, and engaging in information exchange with external actors. Resource Dependence Theory has been applied in a variety of contexts, including healthcare, education, and tourism [44]. In the context of tourism, researchers have used RDT to explore how tourism businesses can effectively manage their relationships with digital intermediaries, such as online travel agencies and search engines. Scholars have also used RDT in order to understand the mechanisms of inter-organizational relationships in the context of tourism [45].

To determine which studies, evaluate e-commerce and tourism together, a bibliometric analysis was conducted. This involves a comprehensive search of relevant databases and journals, using specific keywords and inclusion criteria to identify studies that address the intersection of e-commerce and tourism. The studies can then be analyzed and synthesized to identify the emerged key themes and findings. Moreover, by examining the relationship between e-commerce and tourism, scholars can help to have a better knowledge of social, cultural, and economic impacts of these phenomena. This can help to inform public discourse and policy decisions related to issues such as sustainable tourism development, consumer privacy, and intellectual property rights. Unlike the medical sciences, management (and social sciences in general) have not developed standard, agreed-upon protocols for collecting best-practice evidence [46]. Due to that reason some other methods such as systematic review was not used in this study. Also, visualization tools can be helpful in identifying which ones have had a significant influence on research agendas, when thousands of articles have been published on a subject [47]. Overall, the integration of e-commerce and tourism research has the potential to generate new knowledge and insights that can inform both academia and industry, while also contributing to broader societal debates and challenges.

Bibliometrics is a field of study that uses quantitative methods to analyze the production, dissemination, and impact of scholarly publications. It involves the measurement of various aspects of scientific communication, such as the number of publications, citations, and co-authorships [48,49]. Bibliometrics provides insights into the productivity, influence, and collaboration patterns of researchers, as well as the dynamics of scientific fields and disciplines. The use of bibliometric indicators has become increasingly common in academic evaluation, funding allocation, and research assessment, and has raised important questions about the validity and ethics of such practices [50,51]. As such, bibliometrics is a crucial instrument for understanding the structure and evolution of scientific knowledge [52,53].

The aim of this study about e-commerce and tourism with bibliometrics would be to investigate intellectual production of the effect of research related to these two fields. The study would use bibliometric analysis, which involves the quantitative analysis for citation data and publications. Bibliometric indicators such as citation counts, h-index scores, and co-authorship networks may be used to determine the most influential authors, institutions, and publications in the field, as well as to map the intellectual framework of the field and determine emerging trends and research gaps. By identifying the most influential authors, institutions, and publications, this study can inform academic institutions and researchers about the key players in the field and the most important works that have been published. Moreover, by identifying emerging trends and research gaps, the study can inform future research in this area and help to guide the development of new research agendas.

## 3 Materials and method

As a discipline, it is seen that many related concepts have emerged in the field of bibliometrics, as in many other disciplines. However, it can be stated that the differences between these concepts are not clear. For this reason, the development of bibliometrics should not be considered separately from the development of related concepts. In fact, it is seen that the conceptual development in the field of bibliometrics is realized in the form of statistical bibliography bibliometrics scientometrics informetric [54]. Today, bibliometrics has developed as among the few disciplines of inquiry that are really multidisciplinary research fields that spans practically all scientific disciplines [55]. Pritchard defined bibliometrics as “the application of mathematics and statistical methods to books and other media of communication” [56].

Bibliometrix is an open-source software developed by Massimo Aria and Corrado Cuccurullo. Since it is programmed in the R language, it is flexible, fast-developable, and suitable for integration with other R programs. For this reason, Bibliometrix is considered useful in an ever-changing and evolving field such as bibliometrics. Another feature of Bibliometrix is that it guides the researcher through the bibliometric analysis processes. Another important feature of Bibliometrix is that it can calculate the h-index and its derivatives as performance indicators of authors in terms of performance analysis [57]. In addition to all these, Bibliometrix allows both performance analysis (overview, references, authors, documents) and scientific domain mapping (conceptual structure, intellectual structure and social structure) procedures [58]. Also, Bibliometrix offers a different clustering approach to analyze conceptual structure using factor analysis [59]. Due to all these reasons, authors preferred to use Bibliometrix in this research. The most criticized feature of this software is to enable researchers to use only one database as WoS and Scopus databases use a very different approach to codify the bibliographic metadata meaning that it is not possible to do citation and co-citation analyses on merged databases [60].

We performed a systematic search through the Wos database during in February 2023 according to PRISMA. PRISMA is a minimum database of items required for reporting in meta-analyses and systematic reviews. PRISMA is mostly used for reporting reviews that assess the results of interventions. It can also serve as a foundation for publishing systematic reviews whose goals are different from assessing therapies, like assessing the cause, prevalence, diagnosis, or prognosis. PRISMA is a tool created to help writers report meta-analyses and systematic reviews more effectively [61]. The dataset was obtained from Wos database. According to the statement, WoS is generally better for data quality (standardized reference items, Keywords Plus availability, minimal missing data, etc.). However, Scopus is a superior option if you need to study conference proceedings or publications in the arts and humanities [60]. Despite the fact that Scopus is another major database, several scholars [[62], [63], [64]] state that quality of WoS is still one of the highest ones. Thomson Reuters created the Web of Science as a web based technology in 1960 [65]. Also, there are some other reasons why Web of Science Core Collection database was preferred for data collection in this study. The Web of Science Core Collection contains more than 20,000 items in more than 250 scientific disciplines. It is an important database of more than a dozen peer-reviewed, scientific journals [66]. The most important data in bibliometric research sources are SCI, SSCI, A&HCI scientific citation indexes and these indexes include Web of Science Core Collection database [67,68]. In addition to this, many SSCI&ESCI-indexed and the most-cited bibliometric studies in tourism field used Web of Science Core Collection [[69], [70], [71], [72]].

**Fig. 1:**
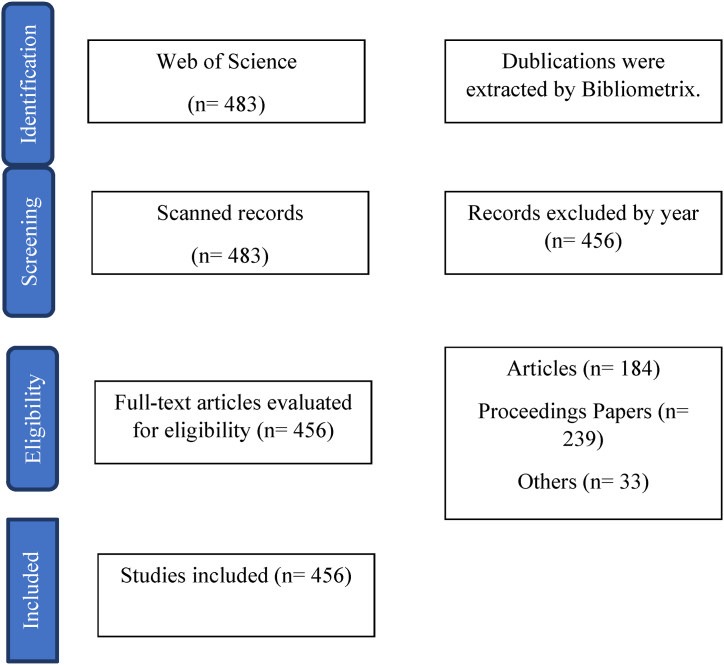
Preferred Reporting Items for Systematic Review and Meta-Analysis (PRISMA) flow chart (adapted from Ref. [76]).

The authors did not wish to include any unrelated papers; therefore, they entered two keywords: “e-commerce” and “tourism”. Keyword searches have become a common search statistic in databases and search engines for information retrieval [73]. People employ keywords to reflect their cognitive understanding of information resources and their content, which is an important means of organizing knowledge and information [74]. Scientific papers, webpages, and multi-media are a few examples of information resources that writers and viewers tag with keywords [75]. The search was initiated by selecting the abstracts. 2023 was not included as the year has not finished. This period enables consideration of the research carried out about “e-commerce” and “tourism.”. Our methodological approach followed the research strategy adopted in prior studies evaluating the contribution provided by scholars to discussion of the e-commerce and tourism. This search (without excluding 2023) retrieved an initial sample of 483 documents. Then, exclusion was applied. The final set of 456 papers was then used for the bibliometric analysis and the systematic review. During this phase, adjustments were made to address errors and inconsistencies in the database, for example, homogenizing the authors’ keywords spelling. Preferred Reporting Items for Systematic Review (PRISMA) flow chart and the criteria detailed further in this study was illustrated in Fig. 1 below.

After all, 456 publication was found, and they were put into process. The main problem that wanted to be solved in this study is to identify the trends in scientific publications on e-commerce and tourism. Also, how institutions, authors, and countries interact with each other in scientific publications related to e-commerce and tourism was wanted to be solved. As there has been no publications analyzing the e-commerce and tourism together in detail in the literature, researchers have decided to answer 6 research questions to solve these main problems. This study aims to provide answers to the research questions which are:

RQ1 What are the bibliometric findings of the dataset including the main information, annual scientific production, and average citation per year?

RQ2 What are the bibliometric findings of the authors including the most relevant authors, author's production over time, and most cited countries?

RQ3 What are the bibliometric findings of the documents including the most global cited documents?

RQ4 What are the bibliometric findings of the conceptual structure including the co-occurrence network, thematic map, thematic evolution, and factorial analysis?

RQ5 What are the bibliometric findings of the intellectual structure including the historiography analysis?

RQ6 What are the bibliometric findings of the social structure including the collaboration network and the collaboration world map?

During the analysis process, the main information, average citation per year, annual scientific production, the most relevant authors, author's production over time, and most cited countries, the most global cited documents, the conceptual structure including the co-occurrence network, thematic evolution, thematic map, and factorial analysis were run.

## 4 Findings

### 4.1 Findings of the dataset

According to the findings, 456 documents were found in 377 different sources such as journals, books, and so on. Another important bibliometric finding which is average years from publication was found as 8,61 and average citations per documents was found as 9,311. When average citations per year per doc is examined, it was found as 1,239. Most of the publications consist of proceeding papers whereas the number of articles was found as 184. 1051 authors were seen 1225 times as authors meaning that some of the authors have contributed more than one paper. Only 89 authors are the materials written solely by one author while 962 authors are multi-authored publications. Also, the number of authors per publication was found as 2,3. The other details are in Table 1.

**Table 1:** Main information about data.

Description	Results
**MAIN INFORMATION ABOUT DATA**	
Timespan	2000:2022
Sources (Journals, Books, etc)	377
Documents	456
Average years from publication	8,61
Average citations per documents	9,311
Average citations per year per doc	1,239
References	13,022
**DOCUMENT TYPES**	
article	184
article; book chapter	4
article; data paper	1
article; early access	5
article; proceedings paper	10
editorial material	1
proceedings paper	239
proceedings paper; retracted publication	1
review	10
review; book chapter	1
**DOCUMENT CONTENTS**	
Keywords Plus (ID)	452
Author's Keywords (DE)	1293
**AUTHORS**	
Authors	1051
Author Appearances	1225
Authors of single-authored documents	89
Authors of multi-authored documents	962
**AUTHORS COLLABORATION**	
Single-authored documents	100
Documents per Author	0,434
Authors per Document	2,3
Co-Authors per Documents	2,69
Collaboration Index	2,7

Source: Own elaboration, done with Bibliometrix software.

**Fig. 2:**
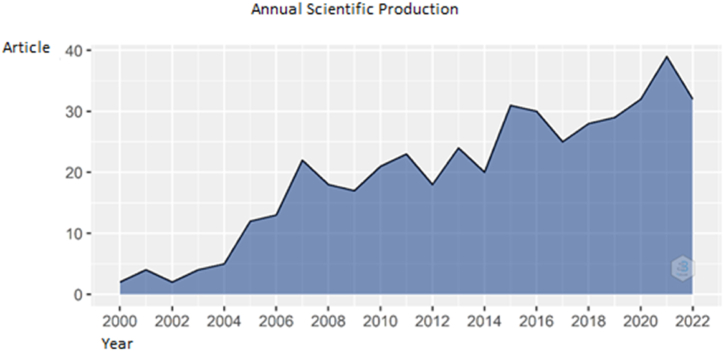
Annual scientific production. Source: Own elaboration, done with Bibliometrix software.

Annual Growth Rate was found as 13.43% in the analysis. The studies have reached its peak point in 2021 with 39 documents. Studies about the related keywords was first seen in 2000 with 2 documents. Publications on the subject became regular in 2007. 2015 is the year when studies reach 31. Fig. 2 shows, there has been a gradual rise in yearly scientific production. Also the number of publications has grown substantially ever since. As it is a growing topic, more studies are expected to have been published in 2023 than in 2022 by looking at Fig. 2.

**Fig. 3:**
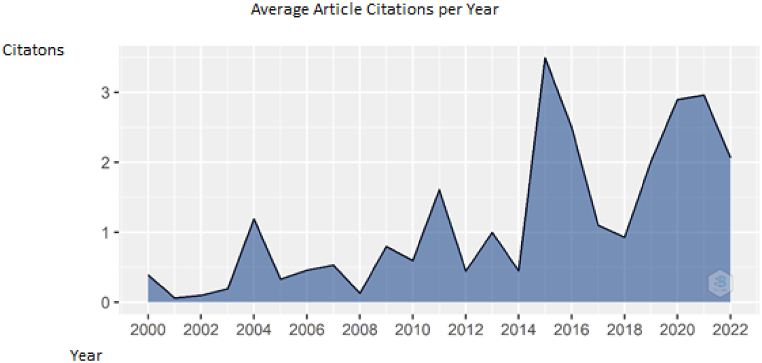
Average publication citations per year. Source: Own elaboration, done with Bibliometrix software.

Unlike the annual scientific production, there is a fluctuation in the figure of average publication citations per year. The citations have reached its peak point in 2015 (n = 3,5). 2016 and 2021 can be classified as 2nd (n = 3) and 2020 can be classified as 3rd (n = 2,9). The other details can be seen in Fig. 3.

### 4.2 Findings of the authors

4 analyses about the authors which are the most relevant authors, author's production over time, and most cited countries were used in this section.

**Fig. 4:**
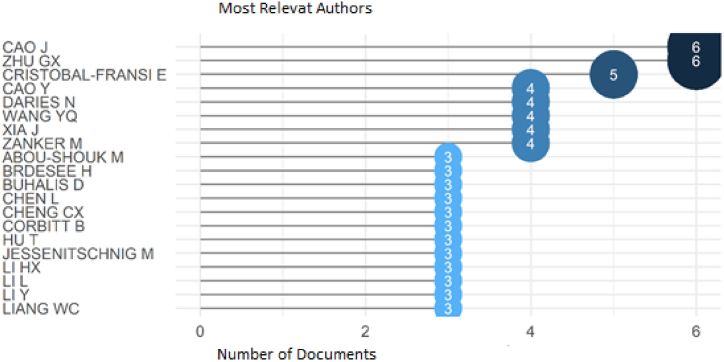
Most relevant authors. Source: Own elaboration, done with Bibliometrix software.

When the most relevant authors analysis was run, 20 of the authors were found as it can be seen in Fig. 4. According to the results, Cao and Zhu have 6 publications whereas Cristobal-Fransi has 5 publications. The details about the other authors can be seen in the figure below. It can be stated if any author wants to publish an article or a proceeding paper or a book chapter about “e-commerce”, and “tourism” should search for the publications of these authors.

**Fig. 5:**
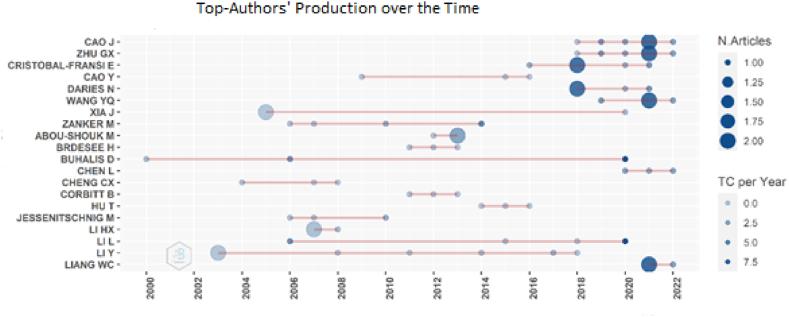
Top-authors' production over time. Source: Own elaboration, done with Bibliometrix software.

When the graphical parameter of this chosen as 20 just like the previous analysis about the authors, Authors' Production over Time was illustrated in Fig. 5 below. The authors who have more than one article have bigger blue dots in the figure. However, the color density changes according to the total citations per year. Therefore, Cao, J., Zhu, G.X., Wang Y.Q. and Liang, W.C. have 2 articles in 2021 with 6,67 total citations per year. Cristobal-Fransi, E. and Daries, N. have 2 articles in 2021 with 6,17 total citations per year. Abou-Shouk, M. have 2 articles 2013 with 2,27 total citations per year. Li, H.X. whom has 2 articles in 2007 with 0,29 total citations per year. Although Xia, J. and Li, Y. have 2 articles, they have no citations.

**Fig. 6:**
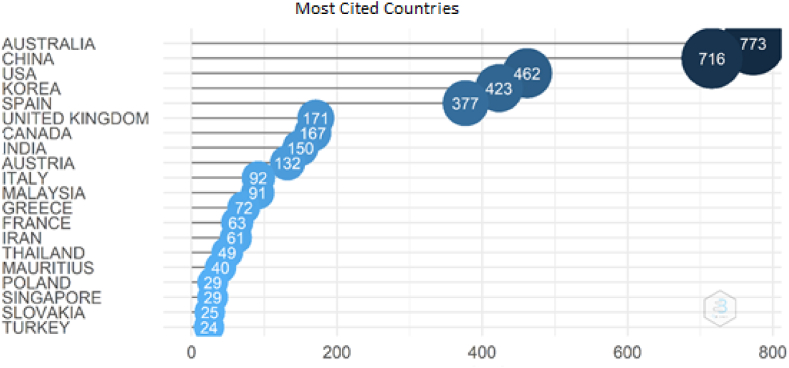
Most cited countries. Source: Own elaboration, done with Bibliometrix software.

Last analysis of the most cited countries in this section was illustrated in Fig. 6. According to the total citations (TC) parameter, Australia and China which are the first two countries have 773 and 716 citations respectively. U.S.A. (TC = 462), Korea (TC = 423), and Spain (TC = 377) follow the first two countries. The other countries which have less than 200 citations can be seen in the figure as well.

### 4.3 Findings of the documents

Globally cited documents, reference spectroscopy, and word dynamics analysis have been applied in this section.

**Fig. 7:**
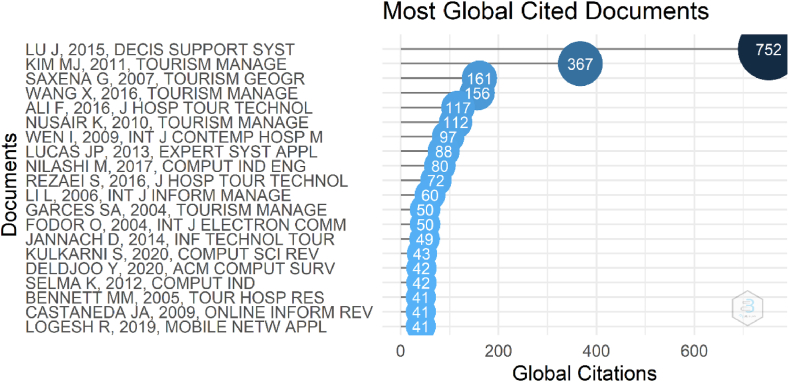
Most global cited documents. Source: Own elaboration, done with Bibliometrix software.

When Fig. 7 “Most Global Cited Documents” is examined below, the paper of [77] titled as “Recommender system application developments: A survey” was ranked as the 1st one and followed by Ref. [78] titled as “*the effect of perceived trust on electronic commerce: Shopping online for tourism products and services in South Korea*”. The figure below contains the other information of the papers and authors whose papers are the Most Global Cited Documents, and which have less than 300 total citations.

### 4.4 Conceptual structure

Bibliometrix has 4 different Conceptual Structure Analysis and 4 fields which are Keywords Plus, Authors Keywords, Titles, and Abstracts. 4 different elements were chosen as a parameter in each analysis in this study. This section and the next 2 sections have the most complicated analysis. Due to that reason, formulas and codes were given to be able to explain the findings in detail. Another research conducted by Koseoglu [70] mentioned two bibliometric methods which are basic and advanced. The first one contains content analysis and metrics to measure the performance of the papers and/or contributors so that researcher can review the publications by using them. On the other hand, the second one contains advanced variety of methods, such as co-occurrence network, thematic evolution, thematic map, and factorial analysis, historiograph analysis as this paper does.

**Fig. 8:**
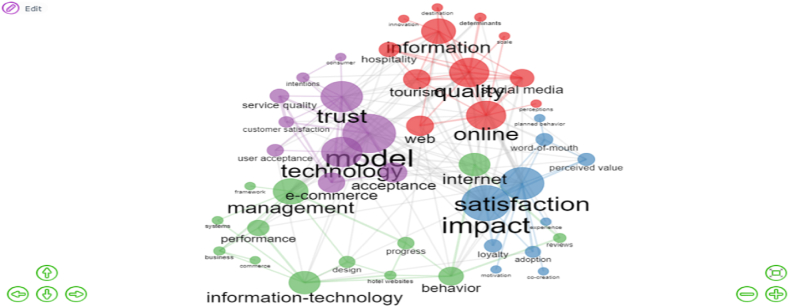
Co-citation network (Parameter: Keywords Plus). Source: Own elaboration, done with Bibliometrix software.

Co-citation happens when two articles are both cited in a third article. As a result, co-citation is the inverse of bibliographic coupling. The following generic formula can be used to generate a co-citation network:

Bcoup = A’ × A

where A is a Document × Cited reference matrix [57]. As shown in Fig. 8, co-citation network identified four clusters in four different colors. 4 clusters which we have identified that we call the management in green cluster (PageRank = 0,043); model in purple cluster (PageRank = 0,058); satisfaction in blue cluster (PageRank = 0,063); quality (PageRank = 0,055) in red cluster. PageRank reflects an article's citations in highly cited publications and measures its overall status in the domain. It is used by researchers to assess the strength of links in the citation network [79]. According to the PageRank, the main keywords in the management cluster are information-technology, behavior, and internet. We found the words trust, technology, acceptance, and e-commerce in the model cluster. Impact is the most dominant keyword in the satisfaction cluster. Finally, online, information, social media, tourism, hospitality, and web are the most eye-catching keywords plus in quality cluster. It's fascinating to observe clusters as a small set of terms reflecting different studying fields. These clusters are evident in the figure below.

**Fig. 9:**
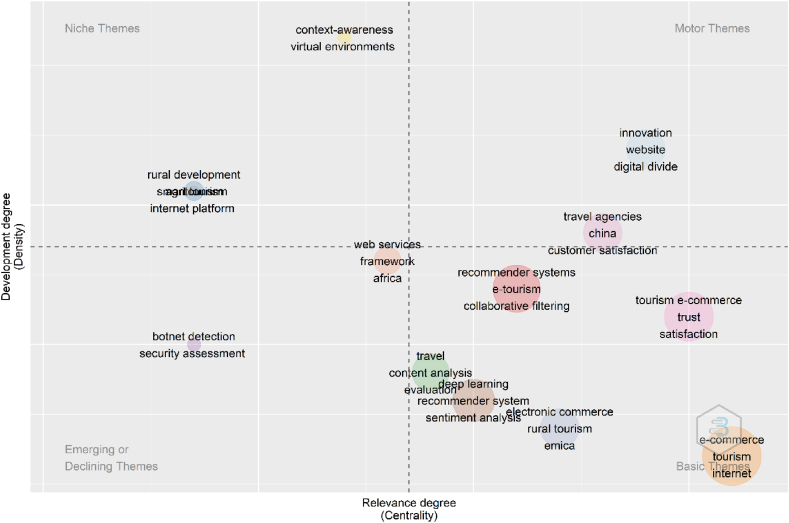
Thematic map (Parameter: Authors Keywords). Source: Own elaboration, done with Bibliometrix software.

According to Ref. [80], scientists and academicians are able to comment on the thematic map in a more easily comprehendible way recently. As seen in Fig. 9, there are 4 quadrants which are going to be described as the first, the second, the third, and the fourth here. While the first one shows which is the central and developed motor themes, the second one which is central and undeveloped shows basic and transversal themes. The third one which is peripheral and developed shows highly developed and isolated themes, whereas the fourth which is peripheral and undeveloped shows emerging or declining themes [81]. The formulization and codes can be summarized as follows: Document × Authors' keyword [A < - cocMatrix (M, Field = “DE”, sep = “; ”)] or Document x Keyword Plus [A < - cocMatrix (M, Field = “ID”, sep = “; ”)] [57]. The major issue among the more developed “motor themes” in the literature is innovation. (occurrences = 6). This theme is related to different concepts, such as website, digital divide, travel agencies, and China in the first quadrant. According to the second quadrant, tourism e-commerce is the most occurred author's keyword among transversal themes. With regard to the upper-left quadrant, it demonstrates high density themes but unimportant external linkages, which are thus of little value for the field [64]. Context-awareness, virtual environments, rural development, smart tourism, agritourism, and internet platform with web services are in this third quadrant. The developing or fading themes are located in the lower-left quadrant [64]. Framework, Africa, botnet detection, and security assessment are the Authors keywords in this quadrant.

**Fig. 10:**
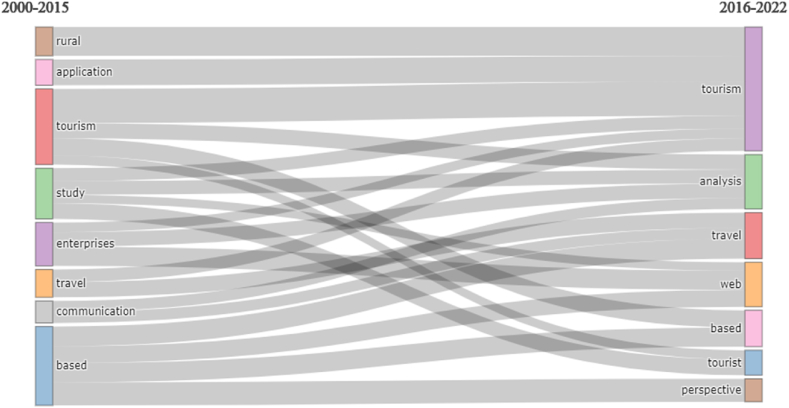
Thematic evaluation (Parameter: Titles). Source: Own elaboration, done with Bibliometrix software.

Fig. 10 shows a diagram depicting the progression of research themes among two different time slices. The first one is from 2000 to 2015 and the second one is from 2016 to 2022. This analysis gives us a frame about the evaluation of titles in publications throughout the world yearly. Each cluster is represented by a node in this analysis and each of them is designated by the first three words of the clusters, Also, the edges shows us the temporal evolution track [[80], [81], [82]]. Also, Fig. 10 exhibits keyword evolution in two stages (2000–2015, and 2016–2022). Keywords “tourism” and “e-commerce” are significant keywords as this indicates in two stages, nevertheless “application”, “rural”, “study”, “enterprise”, “communication”, have only shown up in the first stage. Fig. 10 demonstrates that stage one (2000–2015) and stage two (2016–2022) have a week connection, since there just three keywords that are shared by these two stages.

**Fig. 11:**
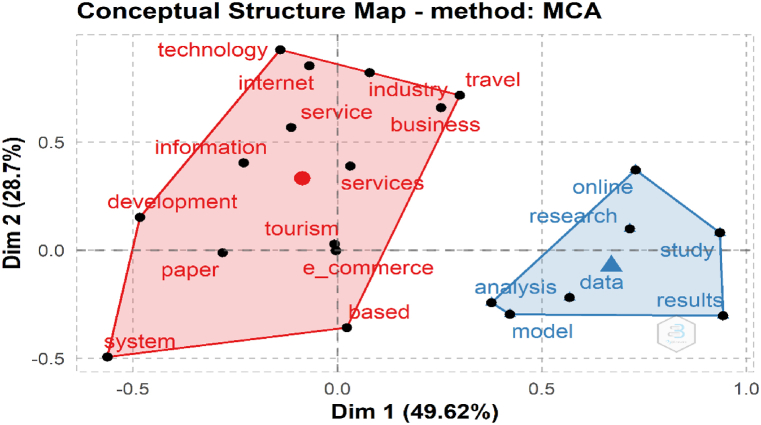
Conceptual structure map (Parameters: Multiple Correspondence Analysis, Abstracts). Source: Own elaboration, done with Bibliometrix software.

The conceptual framework reflects the links between concepts and words in a collection of publications in order to map what science is examining and to investigate the many themes created in research [[83], [84], [85]]. MCA analyzes the homogeneity of an indicator matrix to provide a low-dimensional Euclidean depiction of the initial data [86]. MCA (Multiple Correspondence Analysis) is applied to a Document x Word matrix A in co-word analysis. The words are mapped out in two dimensions [57]. Fig. 11 depicts the abstract analysis's conceptual structure map. The point here reflects terms that appear frequently in abstracts. Closer points highlight a substantial chunk of the content that uses these keywords together. This analysis divides the words into two groups. Cluster 1 (red color) comprises of papers regarding on tourism and technology. This cluster covers keywords such as internet, business, information, tourism, e-commerce, service, and services. Cluster 2 focuses more on academic and technologic keywords such as data and research.

**Fig. 12:**
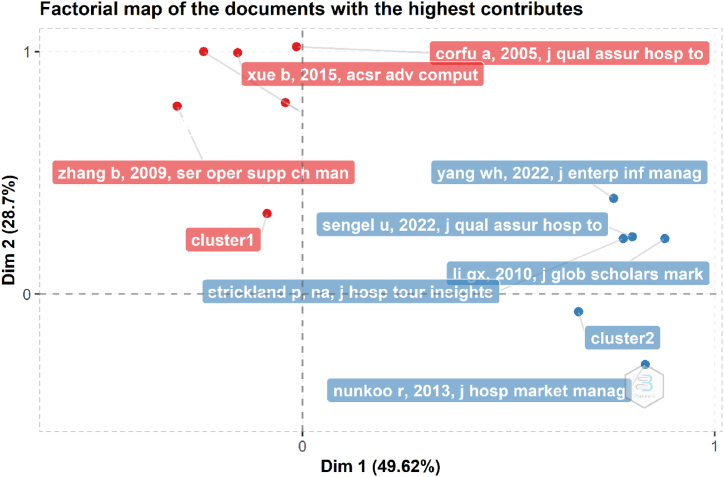
The factorial map showing the publications with the highest contributions. (Parameters: Multiple Correspondence Analysis, Abstracts). Source: Own elaboration, done with Bibliometrix software.

Fig. 12 uses a factorial map to identify the most contributing papers. Because the Bibliometrix - R program supports factorial analysis to establish the conceptual structure of bibliometric data, it provides an intriguing black-box-based option for seeing cluster components based on proximity estimated using the Multiple Correspondence Analysis (MCA). The dimensions or parameters taken into account include keywords, the number of papers per author, and the TC [87]. As shown in the diagram, the papers are sorted into two groups based on two dimensions or criteria. Four papers from Cluster 1 and four documents from Cluster 2 are ranked with the highest contributions since they fall in the positive quadrants of both dimensions.

### 4.5 Intellectual structure

Bibliometrix can also undertake historiographic analysis, as suggested by Ref. [88]. The histPlot function creates a chronological citation network (also known as a historiograph), which is a chronological map of the most relevant citations from a bibliographic collection [57].

**Fig. 13:**
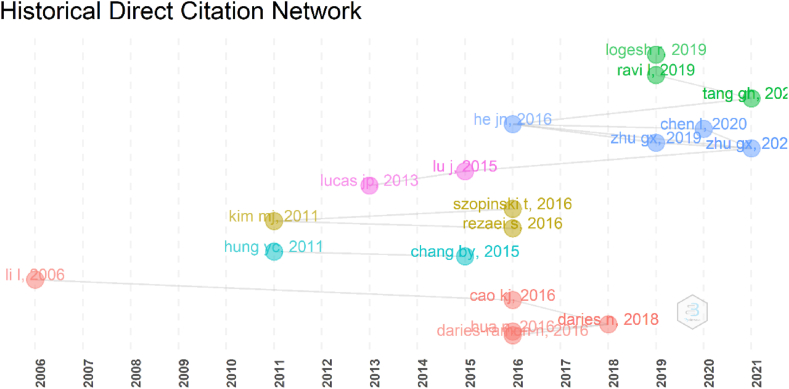
Historiograph (Parameters: Multiple Correspondence Analysis, Abstracts). Source: Own elaboration, done with Bibliometrix software.

A historical direct citation network employs a chronological citation network to create the intellectual structure (Fig. 13). It “*represents a chronological map of the most relevant citations resulting from a bibliographic collection*” [89]. A historiograph is built on direct citations and creates intellectual connections in chronological sequence. The node represents the item mentioned by other documents in the examined collection, the edge denotes direct citation, and the horizontal axis shows the publication years [57]. The intriguing feature of this visualization is not the author/s' names per se, but rather their areas of interest in e-commerce and tourism, as well as the subsequent debate that researchers initiate in the scientific field [64].

Historical path (orange color) represents the e-commerce in tourism and its development over time [2,[90], [91], [92]]. While the turquoise color path identifies application and adoption of e-commerce in tourism [35,36], the yellow color path focuses on parameters such as impulse, trust, and socio economic factors [78,93,94]. Although the application of e-commerce was studied before, the concept of recommendation of e-commerce was developed by the authors in pink color [77,95]. The papers of authors in the blue and green path mostly specializes on a more interdisciplinary view of the terms e-commerce and tourism [96,97].

### 4.6 Social structure

**Fig. 14:**
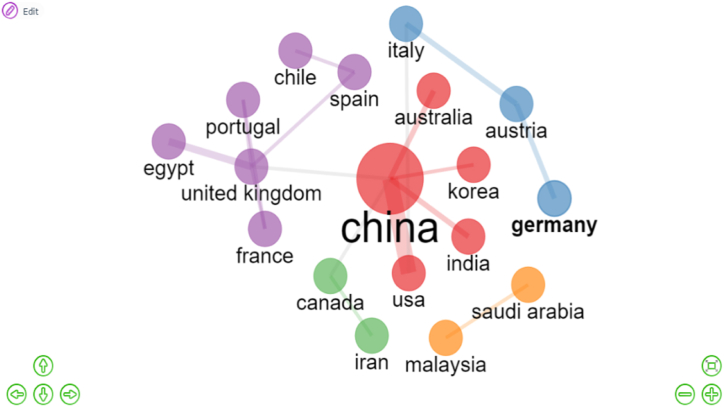
Colloboration network (Parameters: Countries). Source: Own elaboration, done with Bibliometrix software.

**Fig. 15:**
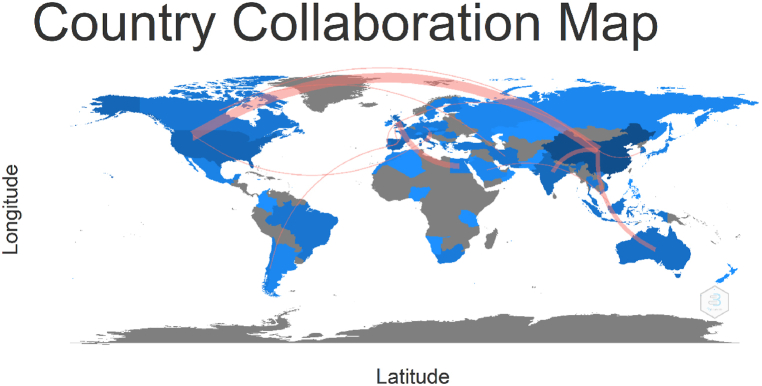
Collaboration world map. Source: Own elaboration, done with Bibliometrix software.

The Social structure of this study consists of 2 analysis which are Colloboration Network and Collaboration World. Document × Country is the general formula for this calculation. However, the nations of the authors are not a typical property of the bibliographic data frame [57]. Fig. 14 shows that China plays a vital role and displays a solid relationship with the USA. Fig. 15 shows the most significant partnership, illustrated by thick lines, and it is between the following pairs: China-USA (f = 9), UK-Egypt (f = 4), China-Australia (f = 3), China-India (f = 3), Italy-Austria (f = 3). 5 clusters were identified in this analysis. China, which is at the heart of the network, works with the majority of countries. China, the United Kingdom, and Austria have demonstrated their ability to serve as bridges between other countries and continents.

## 5 Conclusions

This study tries to understand the trends in the scientific publications about tourism and e-commerce all over the world. Besides, it aims to understand the network among authors, countries, and institution via a bibliometric overview. To be able to perform that, answers of the 6 research questions were found within the scope of the research. According to the results, 456 documents were found in 377 different sources such as journals, books, and so on. Another important bibliometric finding which is average years from publication was found as 8,61 and average citations per documents was found as 9,311. 89 authors are the single-authored documents. On the other hand, 962 authors are multi-authored documents. Annual Growth Rate was found as 13.43% in the analysis. The studies have reached its peak point in 2021 with 39 documents. Studies about the related keywords was first seen in 2000 with 2 documents. Cao, J. and Zhu, G.X. have 6 publications about subject. Cao, J., Zhu, G.X., Wang Y.Q. and Liang, W.C. have 2 articles in 2021 with 6,67 total citations per year. Australia and China which are the first two countries have 773 and 716 citations respectively. During the co-citation network analysis, we identified four clusters which are management, model, satisfaction, and quality. Motor themes in this study are website, digital divide, travel agencies, and China. Keywords “tourism” and “e-commerce” are important keywords as this shows up in two stages of thematic evaluation. Cluster 1 of the Conceptual Structure Map comprises of papers regarding on tourism and technology. This cluster covers keywords such as internet, business, information, tourism, e-commerce, service, and services. Cluster 2 of this map focuses more on academic and technologic keywords such as data and research. Historical path (orange color) represents the e-commerce in tourism and its development over time. Collaboration Network and Collaboration World Map shows that China plays an important role and works closely with the United States.

The number of studies on tourism and e-commerce has been increasing continuously from 2000 to 2022 [30,[98], [99], [100]]. Studies in tourism and e-commerce may attract interest for researchers in the years to come and offer researchers the opportunity to create more impact (e.g. citations). Tourism, e-commerce, trust, satisfaction, recommender system, e-tourism, collaborative filtering, content analysis, deep learning, recommender system these keywords indicate central but not very developed areas of study or concepts. The findings, which we can say will contribute to the researchers’ ability to analyze the domain correctly, show that studies focusing on the keywords mentioned above are needed. We see that the studies, which included the words “communication” [101], “enterprises” [102], “application” [103] and “rural” from the title between 2000 and 2015, decreased during the 2016–2022 period, but the words “analysis” [104], “tourist” [105], “web” [106], “perspective” [107] and “based” appeared more in the titles. Research priorities frequently change over time due to the dynamic nature of the e-commerce and tourism industries. The drop that has been seen could be the result of a shift in research priorities brought about by new developments in technology, shifts in society, or other outside influences. Keywords like “analysis”, “tourist”, “web”, “perspective”, and “based” are becoming more popular, which indicates that new subjects or areas of interest are developing in the field. This change could reflect how e-commerce and tourism are developing, as academics continue to look at new perspectives and facets. We think that the findings are important in terms of giving researchers an idea of what the studies, which focus on the words mentioned above increased during the 2016–2022 period, may become more popular in the future and which new areas may evolve.

Cluster 1 (Conceptual Structure Map): Tourism and Technology (Internet, Business, Information, Services); in this cluster, Resource Dependence Theory suggests that tourism businesses may be dependent on e-commerce platforms and other technologies to access customers and resources. For instance, businesses may rely on online travel agencies (OTAs) like Booking.com or Expedia to reach new customers, or on customer review sites like TripAdvisor to build their reputation. The theory also suggests that this dependence can shape businesses' strategies and decision-making, as they seek to maintain their access to these resources. For example, businesses may need to adapt their marketing strategies to maximize their visibility on OTAs or to respond proactively to negative reviews on customer review sites. The implications for this cluster of our study and Resource Dependence Theory include emphasizing the importance of investing in e-commerce capabilities and developing strategies for leveraging these platforms to their advantage. This might involve developing new ways of engaging with customers online, such as through social media or mobile apps, or investing in data analysis tools to better understand customer preferences and behavior.

Cluster 2 (Conceptual Structure Map): Academic and Technology; in this cluster, Resource Dependence Theory suggests that researchers may be dependent on bibliometric analysis tools and other technologies to access and analyze large datasets. For instance, researchers may use citation analysis tools to discover the most significant journals or authors in their field or use text mining techniques to extract patterns and trends from large datasets. The theory also suggests that this dependence can shape researchers' research questions and methods, as they seek to maximize their access to these resources. For example, researchers may focus on topics that are most amenable to bibliometric analysis or use certain types of data collection methods to optimize their use of text mining tools. The implications for this cluster of our study and Resource Dependence Theory include highlighting the importance of data-driven decision-making in the tourism industry and encouraging businesses to collaborate more closely with academic researchers to develop new insights and innovations. This might involve building partnerships between tourism businesses and academic institutions to share data and expertise or investing in research programs to explore new ways of using technology to improve the customer experience.

Future research can focus on different generations in the field of “e-commerce” and “tourism”. Although studies such as [108] focus on the Y generation, it may be an innovative approach to investigate the behavioral differences of the Y and Z generations in e-commerce [109]. Our research shows that the issue of generational differences has not been examined much in the literature. The findings show that the keywords communication, information, technology, and trust are widely used in tourism and e-commerce research, and future studies can examine consumers' information sharing behaviors [110]. Since it is not possible for consumers in the tourism sector to experience products before purchasing, it is a common behavior for them to benefit from the knowledge of other experienced consumers. Conducting new research on information sharing and sharing commerce in e-commerce platforms of human beings, who are social beings, can be very useful to fill the gap in the literature in the future.

Future studies can use alternative theories to examine this intersection and offer a more thorough understanding. Future research should address the need to investigate the effects of cutting-edge technologies like artificial intelligence (AI), blockchain, and virtual reality on the interaction between e-commerce and tourism. Scopus, which is another well-known database can be used for future studies in the upcoming years. Or combining other databases with Scopus may illuminate the literature. This study ensures that practitioners can obtain practical insights from academic research by explicitly connecting theoretical advances with their applicability in the real world. The paper aims to explicate how academic pursuits in the nexus of e-commerce and tourism might contribute to the making of informed decisions in the domains of business and regulation.

In conclusion, the intersection of e-commerce and tourism has become an increasingly important area of research, as the adoption of e-commerce technologies has transformed the way tourism businesses operate and enhanced the travel experience for consumers. At the same time, bibliometric analysis has emerged as a valuable tool for quantitatively assessing the intellectual production and effect of research related to these fields.

By examining the relationship between e-commerce and tourism scholars can help to have a better knowledge of impact and implications of e-commerce adoption in the tourism industry. Bibliometric analysis can reveal information about the volume and scope of scholarly research related to e-commerce and tourism, as well as the pioneer authors, institutions, and publications for this studying area.

Moreover, by combining bibliometric analysis with other research methods, such as surveys and case studies, scholars can identify emerging trends, research gaps, and opportunities for innovation and growth in the intersection of e-commerce and tourism. This can inform the development of new strategies and business models for tourism businesses, as well as provide insights for policymakers and regulators seeking to facilitate the growth of e-commerce in the tourism sector.

The contribution of studies that evaluate e-commerce and tourism together to the literature can be significant. By examining the interplay between these two fields, scholars can identify new opportunities for innovation and growth in the tourism industry, while also shedding light on the challenges and risks associated with e-commerce adoption. This can inform the development of new business models and strategies for tourism businesses, as well as provide insights for policymakers and regulators seeking to facilitate the growth of e-commerce in the tourism sector.

Overall, the integration of e-commerce and tourism has the potential to generate new knowledge and insights that can inform both academia and industry, while also contributing to broader societal debates and challenges. By continuing to investigate the interplay between these fields, scholars can help to ensure that the tourism industry remains innovative, competitive, and sustainable in the digital age. Specifically, our findings will clarify useful suggestions based on the “Resource Dependence Theory”. To reduce vulnerabilities, this may entail promoting more diverse resource portfolios, encouraging cooperative networks to improve access to essential resources, and creating flexible plans that consider the dynamically shifting dynamics of resource dependency in tourism and e-commerce. We will also stress how important these suggestions are for regulators and politicians. One way to help businesses manage their resource dependencies efficiently is to establish an environment that is supportive of their efforts by incorporating the insights from the “Resource Dependence Theory” into policy considerations. Our paper outlines an approach for future research endeavors as we navigate the complex terrain of resource dependencies. We will push academics to further explore the complex relationships that exist between organizational dependencies and results to further the development of “Resource Dependence Theory” and its relevance to contemporary issues. The conclusions and suggestions derived from our study, which is based on the “Resource Dependence Theory,” not only offer significant direction for decision-makers within organizations but also open new avenues for further investigation and improvement of this theory in the larger scholarly community.

## Limitations

Because the research's conclusions are based solely on a sample from the WOS database, consideration should be taken when applying the findings to more extensive databases or different scenarios. The fact that only WOS data was utilized for the analysis is one of the study's limitations and our research was conducted within a limited timeframe (2000–2022). Resource Dependence Theory could be supported with alternatives.

## Data availability statement

The data that support the findings of this study are available on request from the corresponding author, Ramazan EREN. The data are not publicly available due to restrictions of the Web of Science database.

## CRediT authorship contribution statement

**Mehmet Bahadır Kalıpçı:** Writing – review & editing, Writing – original draft, Resources. **Erkan Kadir Şimşek:** Writing – review & editing, Writing – original draft, Software, Methodology, Data curation. **Ramazan Eren:** Writing – review & editing, Writing – original draft, Visualization, Validation, Resources, Methodology.

## Declaration of competing interest

The authors declare that they have no known competing financial interests or personal relationships that could have appeared to influence the work reported in this paper.

## References

[1] Albadvi A., Saddad G. (2012). Web site evaluation of Iranian tourism and hospitality organizations: an E-commerce web site features model. J. Hospit. Market. Manag..

[2] Hua N. (2016). E-commerce performance in hospitality and tourism. Int. J. Contemp. Hospit. Manag..

[3] Cano J.A., Londoño-Pineda A., Castro M.F., Paz H.B., Rodas C., Arias T. (2022). A bibliometric analysis and systematic review on E-marketplaces, open innovation, and sustainability. Sustain. Switz..

[4] Moro S., Rita P. (2022). Data and text mining from online reviews: an automatic literature analysis. Wiley Interdiscip. Rev. Data Min. Knowl. Discov..

[5] Munar A.M., Jacobsen J.K.S. (2014). Motivations for sharing tourism experiences through social media. Tourism Manag..

[6] Sherer P.D., Suddaby R., Rozsa de Coquet M. (2019). Does resource diversity confer organizational autonomy in arts organizations? Extending resource dependence theory. J. Arts Manag. Law Soc..

[7] Biermann R., Harsch M., Koops J.A., Biermann R. (2017). Palgrave Handb. Inter-organ. Relat. World Polit..

[8] Ku E.C.S. (2022). Technological capabilities that enhance tourism supply chain agility: role of E-marketplace systems, Asia Pac. J. Tour. Res..

[9] Yang Z., Fang X. (2004). Online service quality dimensions and their relationships with satisfaction: a content analysis of customer reviews of securities brokerage services. Int. J. Serv. Ind. Manag..

[10] Schuckert M., Liu X., Law R. (2016). Insights into suspicious online ratings: direct evidence from TripAdvisor, Asia pac. J. Tour. Res..

[11] Yuan Y., Gretzel U., Tseng Y.H. (2015). Revealing the nature of contemporary tourism research: extracting common subject areas through bibliographic coupling. Int. J. Tourism Res..

[12] Paliwal M., Chatradhi N., Singh A., Dikkatwar R. (2022). Smart tourism: antecedents to Indian traveller's decision. Eur. J. Innovat. Manag..

[13] Ranjan J., Foropon C. (2021). Big data analytics in building the competitive intelligence of organizations. Int. J. Inf. Manag..

[14] Franceschet M. (2010). A comparison of bibliometric indicators for computer science scholars and journals on Web of Science and Google Scholar. Scientometrics.

[15] Pavlou P.A. (2003). Consumer acceptance of electronic commerce: integrating trust and risk with the technology acceptance model. Int. J. Electron. Commer..

[16] Holsapple C.W., Singh M. (2000). Toward a unified view of electronic commerce, electronic business, and collaborative commerce: a knowledge management approach. Knowl. Process Manag..

[17] Zott C., Amit R., Donlevy J. (2000). Strategies for value creation in E-commerce: best practice in Europe. Eur. Manag. J..

[18] Berdik D., Otoum S., Schmidt N., Porter D., Jararweh Y. (2021). A survey on blockchain for information systems management and security. Inf. Process. Manag..

[19] Adhikary A., Diatha K.S., Borah S.B., Sharma A. (2021). How does the adoption of digital payment technologies influence unorganized retailers' performance? An investigation in an emerging market. J. Acad. Market. Sci..

[20] Stockdale R. (2007). Managing customer relationships in the self-service environment of e-tourism. J. Vacat. Mark..

[21] Tsai H.T., Huang L., Lin C.G. (2005). Emerging e-commerce development model for Taiwanese travel agencies. Tourism Manag..

[22] Nyarko B., Oppong Mensah N., Boateng K.A., Donkor A. (2022). Influences of E-commerce adoption on sales performance among agrochemical input dealers in the Ghanaian city. Cogent Bus. Manag..

[23] Bilgihan A., Okumus F., Nusair K., Bujisic M. (2014). Online experiences: flow theory, measuring online customer experience in e-commerce and managerial implications for the lodging industry. Inf. Technol. Tourism.

[24] Elliott J. (1987). Government management of tourism — a Thai case study, Tour. OR Manag..

[25] Stone L.S., Nyaupane G.P. (2019). The tourist gaze: domestic versus international tourists. J. Trav. Res..

[26] Sharpley R. (2020). Tourism, sustainable development and the theoretical divide: 20 years on. J. Sustain. Tourism.

[27] Maswera T., Dawson R., Edwards J. (2008). E-commerce adoption of travel and tourism organisations in South Africa, Kenya, Zimbabwe and Uganda, Telemat. Inform.

[28] Rosário A., Raimundo R. (2021). Consumer marketing strategy and e-commerce in the last decade: a literature review. J. Theor. Appl. Electron. Commer. Res..

[29] Buhalis D., Deimezi O. (2004). E-tourism developments in Greece: information communication technologies adoption for the strategic management of the Greek tourism industry, tour. Hosp. Res..

[30] Liu X., Zhang L., Chen Q. (2022). The effects of tourism e-commerce live streaming features on consumer purchase intention: the mediating roles of flow experience and trust. Front. Psychol..

[31] Devece C., Garcia-Agreda S., Ribeiro-Navarrete B. (2015). The value of trust for travel agencies in achieving customers' attitudinal loyalty. J. Promot. Manag..

[32] Vila T.D., González E.A., Vila N.A., Brea J.A.F. (2021). Indicators of website features in the user experience of e-tourism search and metasearch engines. J. Theor. Appl. Electron. Commer. Res..

[33] Martínez-González J.A., Álvarez-Albelo C.D. (2021). Influence of site personalization and first impression on young consumers' loyalty to tourism websites. Sustain. Switz..

[34] Florido-Benítez L. (2023). The role of the top 50 US cargo airports and 25 air cargo airlines in the logistics of E-commerce companies. Logistics.

[35] Chang B.Y., Magobe M.J., Kim Y.B. (2015). E-commerce applications in the tourism industry: a Tanzania case study, South Afr. J. Bus. Manag..

[36] Hung Y.C., Yang Y.L., Yang H.E., Chuang Y.H. (2011). Factors affecting the adoption of e-commerce for the tourism industry in Taiwan, Asia Pac. J. Tour. Res..

[37] Gretzel U., Fuchs M., Baggio R., Hoepken W., Law R., Neidhardt J., Pesonen J., Zanker M., Xiang Z. (2020). e-Tourism beyond COVID-19: a call for transformative research. Inf. Technol. Tourism.

[38] Abou-Shouk M., Megicks P., Lim W.M. (2013). Perceived benefits and E-commerce adoption by SME travel agents in developing countries. J. Hospit. Tourism Res..

[39] Wang J., Wang M., Wu J. (2015). Empirical study on flow experience in China tourism E-commerce market. J. Ind. Eng. Manag..

[40] Morosan C., Hua N., Defranco A. (2017). E-commerce expenses and financial performance of American upper midscale hotels. Tour. Anal.

[41] Lin H.C., Liu X., Huang Y., Chen H.Y. (2022). Determinants of continued use of tourism and hospitality e-commerce platforms and the role of information transparency. Curr. Issues Tourism.

[42] Zhao J., Zhang P. (2023). Investigating the role of E-commerce marketing capabilities to achieve the strategic performance of tourism firms. Front. Psychol..

[43] Pfeffer J., Salancik G. (2003).

[44] Parnell D., May A., Widdop P., Cope E., Bailey R. (2019). Management strategies of non-profit community sport facilities in an era of austerity. Eur. Sport Manag. Q..

[45] Fink R.C., Edelman L.F., Hatten K.J., James W.L. (2006). Transaction cost economics, resource dependence theory, and customer--supplier relationships. Ind. Corp. Change.

[46] Combs J.G., Crook T.R., Rauch A. (2019). Meta‐analytic research in management: contemporary approaches, unresolved controversies, and rising standards. J. Manag. Stud..

[47] Linnenluecke M.K., Griffiths A. (2013). Firms and sustainability: mapping the intellectual origins and structure of the corporate sustainability field. Global Environ. Change.

[48] Thakur P., Khoo C., Pyar W.Y.K. (2021). Diversity training: where are we, and where should we be heading? A systematic literature review. Tour. Recreat. Res..

[49] Toker B., Kalipçi M.B. (2022). Evaluation of sustainable development and travel agencies within the scope of Agenda 2030: a bibliometric analysis, Present Environ. Sustain. Dev..

[50] La L., Xu F., Buhalis D. (2021). Knowledge mapping of sharing accommodation: a bibliometric analysis. Tourism Manag. Perspect..

[51] Şimşek E.K., Kalıpçı M.B. (2022). Education quality and tourism faculty: a bibliometric analysis. J. Tour. Serv..

[52] Yu D., Liu Y., Xu Z. (2023). Analysis of knowledge evolution in PROMETHEE: a longitudinal and dynamic perspective. Inf. Sci..

[53] Yu D., Sheng L., Shi S. (2023). A retrospective analysis of Journal of Forecasting: from 1982 to 2019. J. Forecast..

[54] Qiu J., Zhao R., Yang S., Dong K. (2017).

[55] Glänzel W. (2003). https://www.researchgate.net/publication/242406991_Bibliometrics_as_a_research_field_A_course_on_theory_and_application_of_bibliometric_indicators.

[56] Pritchard A. (1969). Statistical bibliography or bibliometrics?. J. Doc..

[57] Aria M., Cuccurullo C. (2017). bibliometrix: an R-tool for comprehensive science mapping analysis. J. Informetr..

[58] Moral-muñoz J.A., Herrera-viedma E., Santisteban-espejo A., Cobo M.J., Herrera-viedma E., Santisteban-espejo A., Cobo M.J. (2020). 77520-Texto del artículo-249046-3-10-20200304.pdf. El Prof. Inf.- Ción.

[59] Dervis H. (2019). Bibliometric analysis using bibliometrix an R package. J. Scientometr. Res..

[60] Bibliometrix (2024). https://www.bibliometrix.org/home/index.php/faq.

[61] PRISMA (2024). http://www.prisma-statement.org.

[62] Mongeon P., Paul-Hus A. (2016). The journal coverage of Web of Science and Scopus: a comparative analysis. Scientometrics.

[63] Ritchie J.R.B., Crouch G.I. (2000). The competitive destination: a sustainable tourism perspective. Tourism Manag..

[64] Della Corte V., Del Gaudio G., Sepe F., Sciarelli F. (2019). Sustainable tourism in the open innovation realm: a bibliometric analysis. Sustain. Switz..

[65] Sánchez A.D., de la Cruz Del Río Rama M., García J.Á. (2017). Bibliometric analysis of publications on wine tourism in the databases Scopus and WoS. Eur. Res. Manag. Bus. Econ..

[66] Clarivate, Web of Science (2024). https://clarivate.libguides.com/woscc.

[67] Güzeller C.O., Çeliker N. (2018). Bibliometric analysis of tourism research for the period 2007-2016. Adv. Hosp. Tour. Res. AHTR.

[68] Nebioglu O. (2019). Tourism and food consumption: a bibliometric analysis on international literature. J. Travel Hosp. Manag..

[69] Barrios M., Borrego A., Vilaginés A., Ollé C., Somoza M. (2008). A bibliometric study of psychological research on tourism. Scientometrics.

[70] Koseoglu M.A., Rahimi R., Okumus F., Liu J. (2016). Bibliometric studies in tourism. Ann. Tourism Res..

[71] Mulet-Forteza C., Genovart-Balaguer J., Mauleon-Mendez E., Merigó J.M. (2019). A bibliometric research in the tourism, leisure and hospitality fields. J. Bus. Res..

[72] Ülker P., Ülker M., Karamustafa K. (2023). Bibliometric analysis of bibliometric studies in the field of tourism and hospitality. J. Hospit. Tour. Insights.

[73] Ghanbarpour A., Naderi H. (2019). A model-based method to improve the quality of ranking in keyword search systems using pseudo-relevance feedback. J. Inf. Sci..

[74] Chen Y., Ke H. (2014). A study on mental models of taggers and experts for article indexing based on analysis of keyword usage. J. Assoc. Inf. Sci. Technol..

[75] Lu W., Liu Z., Huang Y., Bu Y., Li X., Cheng Q. (2020). How do authors select keywords? A preliminary study of author keyword selection behavior. J. Informetr..

[76] Aşik Z., Özen M. (2019). Meta-analysis steps and reporting. Turk. J. Fam. Med. Prim. Care.

[77] Lu J., Wu D., Mao M., Wang W., Zhang G. (2015). Recommender system application developments: a survey, Decis. Support Syst.

[78] Kim M.J., Chung N., Lee C.K. (2011). The effect of perceived trust on electronic commerce: shopping online for tourism products and services in South Korea. Tourism Manag..

[79] Ding Y., Yan E., Frazho A., Caverlee J. (2010). PageRank for ranking authors in Co-citation networks. J. Am. Soc. Inf. Sci. Technol..

[80] Cobo M.J., López-Herrera A.G., Herrera-Viedma E., Herrera F. (2011). An approach for detecting, quantifying, and visualizing the evolution of a research field: a practical application to the Fuzzy Sets Theory field. J. Informetr..

[81] Yu J., Muñoz-Justicia J. (2020). A bibliometric overview of twitter-related studies indexed in web of science. Future Internet.

[82] Rashid S., Rehman S.U., Ashiq M., Khattak A. (2021). A scientometric analysis of forty-three years of research in social support in education (1977–2020). Educ. Sci..

[83] Aria M., Misuraca M., Spano M. (2020). Mapping the evolution of social research and data science on 30 Years of social indicators research. Soc. Indicat. Res..

[84] Tijssen R.J.W., Van Raan A.F.J. (1989). Mapping co-word structures: a comparison of multidimensional scaling and leximappe. Scientometrics.

[85] Palácios H., de Almeida M.H., Sousa M.J. (2021). A bibliometric analysis of trust in the field of hospitality and tourism. Int. J. Hospit. Manag..

[86] Gifi A. (1990). https://books.google.com.tr/books?id=XkamAAAAIAAJ.

[87] Ghosh A., Satya Prasad V.K. (2021). Off-grid solar energy systems adoption or usage - a bibliometric study using the bibliometrix R tool. Libr. Philos. Pract..

[88] Garfield E. (2004). Historiographic mapping of knowledge domains literature. J. Inf. Sci..

[89] Borgman C.L. (2002). Communication and collaboration scholarlv communication and bibliometrics. Annu. Rev. Inf. Sci. Technol..

[90] Li L., Buhalis D. (2006). E-Commerce in China: the case of travel. Int. J. Inf. Manag..

[91] Daries-Ramon N., Cristobal-Fransi E., Martin-Fuentes E., Marine-Roig E. (2016). E-commerce adoption in mountain and snow tourism: analysis of ski resorts web presence through the eMICA model. Cuad. Tur..

[92] Cao K., Yang Z. (2016). A study of e-commerce adoption by tourism websites in China. J. Destin. Market. Manag..

[93] Rezaei S., Ali F., Amin M., Jayashree S. (2016). Online impulse buying of tourism products: the role of web site personality, utilitarian and hedonic web browsing. J. Hosp. Tour. Technol..

[94] Szopiński T., Staniewski M.W. (2016). Socio-economic factors determining the way e-tourism is used in European Union member states. Internet Res..

[95] Lucas J.P., Luz N., Moreno M.N., Anacleto R., Almeida Figueiredo A., Martins C. (2013). A hybrid recommendation approach for a tourism system. Expert Syst. Appl..

[96] Chen L., Wu Z., Cao J., Zhu G., Ge Y. (2020). Travel recommendation via fusing multi-auxiliary information into matrix factorization. ACM Trans. Intell. Syst. Technol..

[97] Ravi L., Subramaniyaswamy V., Vijayakumar V., Chen S., Karmel A., Devarajan M. (2019). Hybrid location-based recommender system for mobility and travel planning. Mob. Netw. Appl..

[98] Li S., Zhu B., Yu Z. (2023). The impact of cue-interaction stimulation on impulse buying intention on virtual reality tourism E-commerce platforms. J. Trav. Res..

[99] Machová R., Korcsmáros E., Esseová M., Marča R., Changing Trends of Shopping Habits and Tourism During the Second Wave of COVID-19 – International Comparison: Reference: Machová, R., Korcsmáros, E., Esseová, M., Marča R. (2021). Changing trends of shopping habits and tourism during the second wave of COVID-19 – international comparison. J. Tourism Serv..

[100] Xie C., Yu J., Sam S., Huang, Zhang J. (2022). Tourism e-commerce live streaming: identifying and testing a value-based marketing framework from the live streamer perspective. Tourism Manag..

[101] Toma I., Fensel D., Oberhauser A., Fuchs C., Stanciu C., Larizgoitia I. (2013). 2013 IEEE 10th Int. Conf. E-Bus. Eng..

[102] Nugroho H., Ika A., E-Commerce (2016). Proc. Asia Tour. Forum 2016 - 12th Bienn. Conf. Hosp. Tour. Ind. Asia.

[103] Radescu R., Vlad O.-M. (2020). 2020 Int. Symp. Fundam. Electr. Eng. ISFEE.

[104] Zaidan E. (2017). Analysis of ICT usage patterns, benefits and barriers in tourism SMEs in the Middle Eastern countries: the case of Dubai in UAE. J. Vacat. Mark..

[105] Pinto A.S., Costa E., Borges I., Silva F., Abreu A., Rocha Á., Abreu A., De Carvalho J.V., Liberato D., González E.A., Liberato P. (2020). Adv. Tour. Technol. Smart Syst..

[106] Cristobal-Fransi E., Daries N., Serra-Cantallops A., Ramón-Cardona J., Zorzano M. (2018). Ski tourism and web marketing strategies: the case of ski resorts in France and Spain. Sustainability.

[107] Hou Z., Wang P., Chen Z., Luo Y. (2021). Mapping hotspots and emerging trends of tourism E-commerce: a multidisciplinary perspective. Knowl. Organ..

[108] Nadeem W., Juntunen M., Shirazi F., Hajli N. (2020). Consumers' value co-creation in sharing economy: the role of social support, consumers' ethical perceptions and relationship quality. Technol. Forecast. Soc. Change.

[109] German Ruiz-Herrera L., Valencia-Arias A., Gallegos A., Benjumea-Arias M., Flores-Siapo E. (2023). Technology acceptance factors of e-commerce among young people: an integration of the technology acceptance model and theory of planned behavior. Heliyon.

[110] Aparicio M., Costa C.J., Moises R. (2021). Gamification and reputation: key determinants of e-commerce usage and repurchase intention. Heliyon.

